# Innovative Method of Traction in a Bilateral Diaphyseal Femur Fracture in a Polytrauma Below-Knee Amputee

**DOI:** 10.1155/2019/8691398

**Published:** 2019-03-24

**Authors:** Stefan Mitrasinovic, Georgios Kiziridis, Shauni Wellekens, Charline Roslee, Syed Neshat Anjum

**Affiliations:** ^1^Department of Orthopaedics, Southampton General Hospital, Tremona Road, Southampton, UK; ^2^University College London Medical School, Gower Street, London, UK

## Abstract

While diaphyseal femoral shaft fractures are common, it is uncommon to see this injury in leg amputees. Traditionally, these fractures are internally fixed using a fracture table with reduction obtained by traction and adequate rotation exerted on a slightly abducted extremity. Special considerations need to be given in the management of patients with leg amputations. We report the case of a 24-year-old gentleman with bilateral diaphyseal femoral shaft fractures and a previous right below-knee amputation, who was transferred to our centre following a road traffic collision. We highlight important planning that needs to be undertaken for appropriate positioning, ease of reduction, and fracture fixation. We have reviewed the literature to highlight the methods that have been previously described and our use of skeletal traction through the amputation stump that can be utilised by other surgeons in challenging situations like this.

## 1. Background

Bilateral femoral diaphyseal fractures form a good proportion of the trauma and orthopaedic case load and are most commonly due to high-energy trauma, in particular road traffic collisions (RTCs). Due to the aetiology of these fractures, they are often associated with multisystem trauma [1] and thus have a high risk of complications [2]. Preoperative planning is a key in these patients; considerations must be taken for the ideal positioning and surgical approach in order to mitigate nonunion and malunion, which can result in substantial impact on future mobility of the patient [2, 3].

Traditionally, the treatment of choice for a femoral diaphysis fracture is intramedullary nailing with the use of a fracture table. Reduction of the fracture and subsequent internal fixation is achieved by applying axial traction and rotation in a slightly abducted limb [1]. Adequate reduction can be assessed with preoperative imaging studies of the contralateral extremity, providing a reference for length, alignment, and rotation [4]. Patients with an amputated limb and bilateral diaphyseal femur fractures present a unique challenge for the operating team, and conventional techniques are no longer appropriate.

We present a rare case of a bilateral diaphyseal femur fractures in a patient with a preceding right-sided below-knee amputation (BKA) following a polytrauma. This case provides a reference for important preoperative considerations and the unique challenges for this patient group.

## 2. Case Presentation

**Figure 1:**
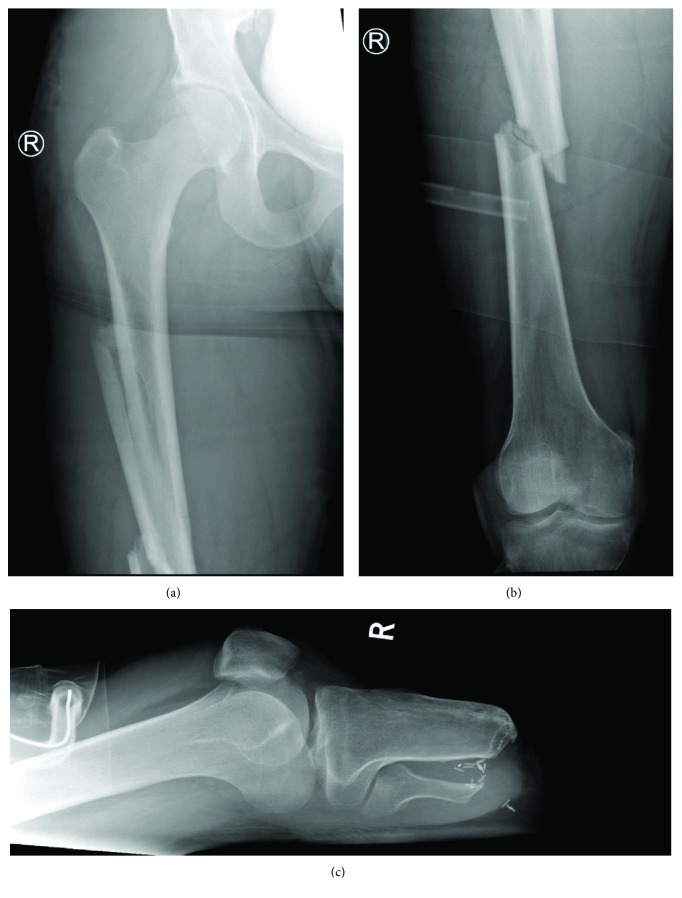
Standard anteroposterior radiograph of the right hip and femur (a), anteroposterior radiograph of the distal right femur (b), and standard lateral radiograph of the right knee (c).

**Figure 2:**
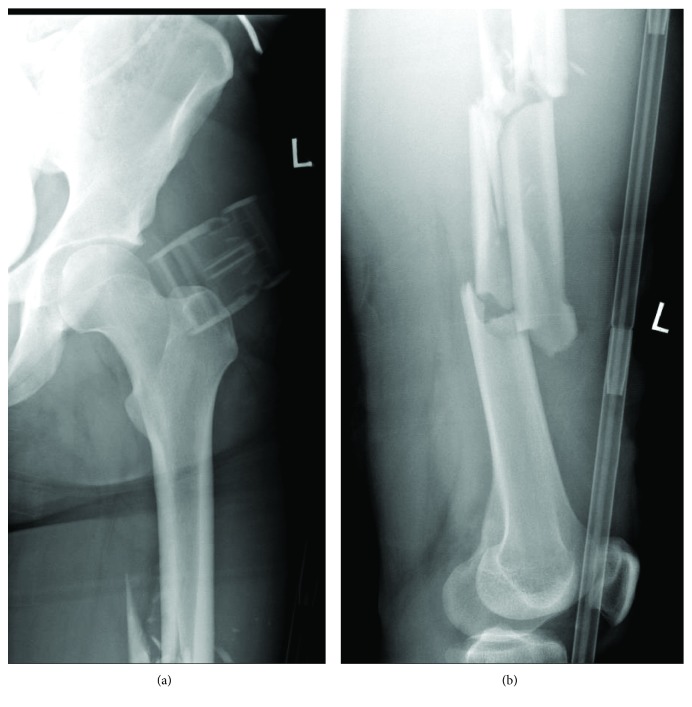
Standard anteroposterior radiograph of the left hip and femur (a); standard lateral radiograph of the left femur and knee (b).

A 24-year-old gentleman was presented to the emergency department following a level 1 trauma call for a high-speed road traffic collision (RTC) car vs. car. The patient had a computed tomography (CT) scan of his head, whole spine, chest, abdomen, and pelvis. His injuries included a left occipital condyle fracture, open displaced transverse fracture of the distal diaphysis of the left humerus, multifragmentary oblique fracture of the middiaphysis of the right femur (Figure 1), displaced multifragmentary fracture of the middiaphysis of the left femur (Figure 2), displaced right transverse process fractures in L3, L4, and L5, and a right pneumothorax.

The patient had a preceding right BKA from a previous traumatic injury and a past medical history of illicit drug use and steroid abuse. The patient did not take any regular medications and did not have any other comorbidities.

## 3. Treatment

The gentleman was initially stabilized and intubated prior to transfer to a tertiary centre. The patient had further treatment in the emergency department with 3 units of red blood cells, 4 units of fresh frozen plasma, and 1 gram of tranexamic acid and subsequently transferred to the intensive care unit (ICU) for inotropic support.

**Figure 3:**
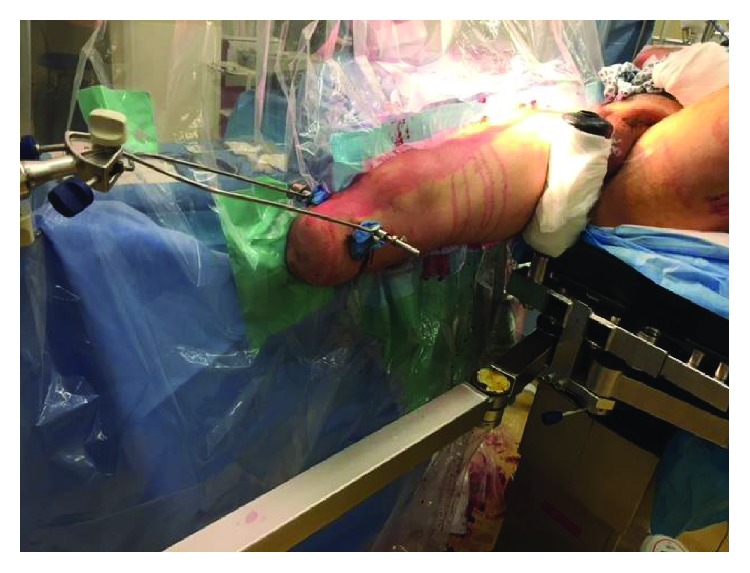
Clinical photograph of skeletal traction achieved by the infracondylar Steinmann pin to the traction device of the fracture table.

The patient was started on intravenous Co-amoxiclav as per our local open fracture protocol due to the open humeral fracture and transferred from ICU to theatre with spinal precautions and a Miami J Collar. He was initially placed on a fracture table for application of the Hoffmann III external fixator to the left femur that helped in positioning of the left leg to aid fluoroscopy access for insertion of the intramedullary nail in the right femur with the amputation stump. Minimally invasive skin incisions were made, and a proximal tibial Steinmann pin (5 mm diameter and 9 inches long) was inserted into the right BKA stump by hand under sterile conditions. A Bohler stirrup was attached to this for traction during ipsilateral femoral nailing. The patient was subsequently transferred to a traction table. Traction, rotation, and slight abduction were then applied under fluoroscopic control through the Bohler stirrup secured to the traction device at the foot end of the table, in order to obtain adequate and stable reduction of the fracture (Figure 3).

**Figure 4:**
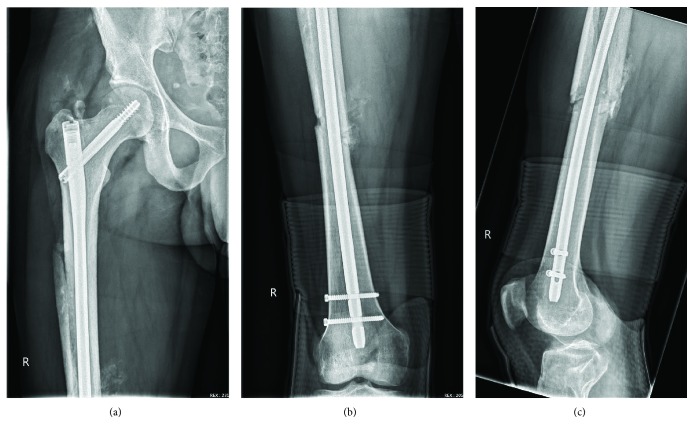
Postoperative anteroposterior radiograph of the right femur (a, b); postoperative lateral radiograph of the right knee (c).

**Figure 5:**
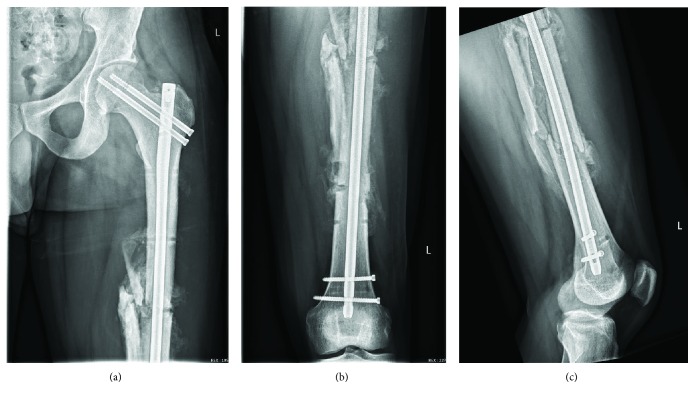
Postoperative anteroposterior radiograph of the left femur (a, b); postoperative lateral radiograph of the left knee (c).

The standard approach to intramedullary nailing was used, with a guidewire inserted through the entry point in the greater trochanter of the femur into the distal fragment of the femur after closed reduction. The nailing was performed after serial reaming of the intramedullary canal. Copious irrigation was performed, and the wounds were closed. The Steinmann pin was removed, and staples were used for the skin closure. Nonadhesive sterile dressings were applied, and no drains were used. The patent's legs were well perfused, and the popliteal pulse was present. Postoperative radiographs showed adequate fracture reduction and fixation (Figures 4 and 5).

The left open fracture of the distal humerus (Gustilo grade 3A) was treated by wound debridement and splinting in above elbow plaster. The definitive fixation of the left femur and left open humerus fracture was delayed due to haemodynamic instability of the patient during surgery.

Once haemodynamically stable, the patient had further surgery 4 days later for removal of the external fixator and intramedullary nailing of the left femur. Open reduction and internal fixation of the left distal humerus and wound closure were carried out in the same operation. The patient's occipital condyle fracture was suitable for conservative management with a Miami J Collar for 6 weeks.

The patient recovered well without any major surgical complications. The patient was refitted with a prosthetic limb and discharged back to his local community hospital for ongoing care.

## 4. Discussion

Diaphyseal femoral shaft fractures are common in the general population but are not frequently reported for patients who have had distally amputated lower limbs; incidence is reported as less than 3% [5, 6]. Special challenges are presented in the operative management of below-knee amputee patients who require internal fixation of their bilateral femoral fractures. Techniques described in orthopaedic textbooks [7, 8] and conventional techniques reviewed for fracture reduction [1] may not be applicable to this patient cohort. Following a review of the literature, we were only able to find one similar case described by Gamulin and Farshad, who presented a patient with a left-sided BKA and a periprosthetic femoral shaft fracture on the same side [9].

Due to the complexity of primary trauma and concomitant injuries, orthopaedic surgeons must be wary of the actions they take in the acute phase. Generally, early reduction and internal fixation are beneficial for better functional outcomes; however, life-threatening concomitant injuries can postpone an immediate operation. Optimal timing for surgery in polytrauma patients can be guided according to the “Damage Control Orthopaedics' principles” [10].

Patients with BKA pose a special problem as positioning them on the fracture table is difficult due to the absence of the foot and part of the lower leg. The problem is accentuated when there is a need to apply traction for adequate reduction of the fracture. There is little information in the literature on techniques to deal with this problem, specifically for diaphyseal fractures in amputees; however, if we broaden the scope of our search we can include other cases involving intertrochanteric fractures of the femur in amputees that provide valuable information about operative technique.

If the fracture is not displaced and no traction is required, then a radiolucent leg support can be used, as described by Rethnam et al. in a case of a bilateral BKA who sustained a right-sided intertrochanteric fracture [11]. Another approach is to fit an inverted traction boot onto the stump, which can allow for some manipulation [11, 12]; however, this requires the stump to be at least 12 centimetres below the knee joint [12]. If traction is required, skin traction should be considered initially and can be applied directly to the stump with adhesive tape and a crepe bandage attached to a traction device on the fracture table [13, 14]. Otherwise, rigid fixation can be applied with a Steinmann pin for accurate control of the fracture in all planes; Berg and Bhatia described using skeletal traction in a left neck of femur fracture in a bilateral amputee (right BKA, left above-knee amputation) by placing the pin in the distal femur of the fractured side and removing the table base to allow for imaging [15]. While not previously reported for amputees, rigid fixation can be facilitated with an AO distractor. The use of the AO distractor has been particularly useful for polytrauma cases in which concomitant injury precludes the initial use of a fracture table [16, 17].

The techniques highlighted above must be balanced for their ability to control rotation or traction forces applied onto the amputated limb until definitive internal fixation is performed [11, 14]. While skeletal traction provides the most control, it provides risk of injury to the soft tissues around the stump, infection, and pull out in the case of osteopenic bone. Chronic skin scar discomfort and pain are an important consideration for the patient, particularly as it may apply to areas of high compressive and shearing forces related to the prosthetic device and thus impacting functional recovery [18]. We did not experience any of these complications in our case and neither have authors of similar case studies [13, 19].

Infections originating from pin sites are a well-described complication [20, 21], and the utmost care should be taken to maintain sterile conditions in the operating theatre. In our case, the Steinmann pin was inserted using minimally invasive stab incisions and left in place for no more than the duration of the procedure, thus minimizing the risk of subsequent infection.

Pull out of the Steinmann pin may occur in patients with degenerative bone conditions such as osteopenia or osteoporosis [11, 14, 19, 22]. Osteoporosis of the stump is a relative contraindication for the insertion of Steinman pin through the stump to provide traction during fracture reduction. This should always be considered prior to use of the device. Those amputees who are load-bearing in the prosthesis generally maintain good bone density. This did not occur in our patient as he was relatively young and was load-bearing through the stump with a prosthesis.

There are other options available for managing challenging cases of bilateral femoral fractures with concurrent below-knee amputations. Femoral distracter can be used to assist reduction and fixation of fracture or other fixation method such as diaphyseal plating could be considered [17]. We chose to stabilize the fracture using intramedullary fixation device that provides better mechanical stability and less soft tissue disruption to aid fracture healing.

## 5. Conclusion

This case demonstrates one of the ways of planning and achieving fixation of bilateral femoral fractures in a patient with a below-knee amputation. Multiple considerations need to be taken for the appropriate time for surgery and method of reduction and subsequent fixation. When evaluating previously published cases, we can affirm that skeletal traction was suitable and necessary for a good surgical and functional outcome. Surgeons should be aware of the other methods presented here to achieve successful closed reduction.

## Abbreviations

BKA: Below-knee amputation
CT: Computed tomography
DHS: Dynamic hip screw
ICU: Intensive care unit
MRI: Magnetic resonance imaging
RTC: Road traffic collision.
